# Hydrophobic binding peptide-conjugated hybrid lipid-mesoporous silica nanoparticles for effective chemo-photothermal therapy of pancreatic cancer

**DOI:** 10.1080/10717544.2017.1396382

**Published:** 2017-11-03

**Authors:** Raj Kumar Thapa, Hanh Thuy Nguyen, Milan Gautam, Aarajana Shrestha, Eung Seok Lee, Sae Kwang Ku, Han-Gon Choi, Chul Soon Yong, Jong Oh Kim

**Affiliations:** a College of Pharmacy, Yeungnam University, Gyeongsan, Gyeongsanbuk-do, Republic of Korea;; b College of Korean Medicine, Daegu Haany University, Gyeongsan, South Korea;; c College of Pharmacy, Hanyang University, Sangnok-gu, Ansan, Republic of Korea

**Keywords:** Bortezomib, hydrophobic-binding peptide, IR-820, pancreatic cancer, chemo-phototherapy

## Abstract

Nanoparticle-based drug delivery systems are designed to reach tumor sites based on their enhanced permeation and retention effects. However, a lack of interaction of these nanoparticles with cancer cells might lead to reduced uptake in the tumors, which might compromise the therapeutic efficacy of the system. Therefore, we developed bortezomib and IR-820-loaded hybrid-lipid mesoporous silica nanoparticles conjugated with the hydrophobic-binding peptide, cyclosporine A (CsA), and referred to them as CLMSN/BIR. Upon reaching the tumor site, CsA interacts hydrophobically with the cancer cell membranes to allow effective uptake of the nanoparticles. Nanoparticles ∼160 nm in size were prepared and the stability of IR-820 significantly improved. High cellular uptake of the nanoparticles was evident with pronounced apoptotic effects in PANC-1 and MIA PaCa-2 cells that were mediated by the chemotherapeutic effect of bortezomib and the photothermal and reactive oxygen species generation effects of IR-820. An *in vivo* biodistribution study indicated there was high accumulation in the tumor with an enhanced photothermal effect in PANC-1 xenograft mouse tumors. Furthermore, enhanced antitumor effects in PANC-1 xenograft tumors were observed with minimal toxicity induction in the organs of mice. Cumulatively, these results indicated the promising effects of CLMSN/BIR for effective chemo-phototherapy of pancreatic cancers.

## Introduction

Pancreatic cancer is the seventh leading cause of cancer-related death in the world and has an estimated 5-year survival rate of <5% (Ilic and Ilic, 2016). Resistance can develop to chemotherapeutic agents, including gemcitabine (used as first-line therapy) (Mini et al., 2006; Jemal et al., 2010) The ineffectiveness of current treatments has left surgery as the main option, thereby necessitating the development of new and effective therapies (Kleeff et al., 2007). Combination therapy is a potential viable approach for the treatment of pancreatic cancer. The combination of chemo- and photo-therapy would treat the tumor by involving different synergistic pathways (DNA damage, ROS generation, and photothermal ablation), leading to cancer cell apoptosis (Wang et al., 2014). Bortezomib (Bor) is a proteasomal inhibitor that causes the accumulation of ubiquitinated proteins in cancer cells, which leads to apoptosis (Thapa et al., 2017). IR-820 is a near infrared (NIR) dye that, upon NIR irradiation, induces photothermal and photodynamic effects, causing cancer cell apoptosis (Fernandez-Fernandez et al., 2012; Fernandez-Fernandez et al., 2014; Zhou et al., 2016). Therefore, a combination of chemotherapeutic agents and NIR dye may effectively cause cancer cell apoptosis and enhance treatment success.

Nanoparticles are drug delivery carriers that have been widely studied for their potential to target the delivery of chemotherapeutic agents to cancer cells (Choi et al., 2016, 2017) Liposomes are well-established drug delivery carriers that are available in several marketed chemotherapeutic formulations owing to their potential for carrying both hydrophilic and hydrophobic drugs, resulting in superior *in vivo* performance (Zhang et al., 2008). Mesoporous silica nanoparticles (MSNs) are promising nanocarrier systems because they have large surface areas and pore volumes, which allow for high drug loading. In addition, the size and pore diameter of MSNs can be varied, which allows for easy functionalization, and MSNs are biocompatible (Castillo et al., 2017). However, the surface of the prepared MSNs is not PEGylated, which might compromise blood circulation time mediated by macrophage opsonization (Thapa et al., 2015). When PEG is on the surface, it protects the hybrid nanosystems and allows for longer circulation times, thereby increasing cumulative delivery to the tumor site (Thapa et al., 2017). Furthermore, functionalized PEG on the surface of liposomes can be utilized for combining different types of ligands for effective nanoparticle delivery to the target site (Biswas et al., 2011; Kim, 2016; Yao et al., 2016) Therefore, a hybrid lipid-MSN system was developed with the aim of enhancing the overall biocompatibility, drug delivery pattern, and resulting anti-cancer effects of the combination of Bor and IR-820 in pancreatic cancer cells.

Ligand- or antibody-conjugated nanocarriers exhibit promising effects only in certain types of cancers that highly express the correlated specific receptors (Li et al., 2014; Vyas et al., 2014). However, the pattern of receptor expression is diverse among different types of tumors and sometimes even within the same tumor, which limits the application of ligand- or antibody-based drug delivery systems. In contrast, another important mechanism, based on the enhanced permeation and retention (EPR) effect, can assist in the passive accumulation of nanoparticles in all types of tumors (Jia et al., 2013). Nanoparticles may reach the tumors; however, due to a lack of interaction with cell membranes, they may be carried away from the tumor site. Therefore, agents that interact with cell membranes would assist in the intracellular uptake of nanoparticles that reach the tumor cells via the EPR effect.

Cell penetrating peptides (CPPs), such as HIV-Tat peptide, penetratin, and transportan, can directly interact with cell membranes via an electrostatic effect and could facilitate nanoparticle intracellular delivery (Bolhassani, 2011). However, these CPPs exhibit a positive charge that induces toxicity in normal cells (Saar et al., 2005; Kilk et al., 2009). Therefore, any interaction between the cells and related molecules that may facilitate the nanoparticle delivery to cancer cells, without causing toxicity to normal tissues, would be an interesting area of study. Hydrophobic interaction between peptides and cell membranes could be utilized as a viable alternative that could act as a driving force for diffusion across membranes (Meyer et al., 2006). Such an interaction is prevalent in several physiological events, including cell signaling, cytolysis, and cellular recognition. In addition, several chemically conjugated hydrophobic-binding peptides (HBPs) have been found effective for delivering cargo inside the cells based on this mechanism (Watkins et al., 2009; Jones and Sayers, 2012; Cai et al., 2014). Cyclosporine A (CsA) is an HBP approved by the FDA for the prevention of graft-versus-host disease that is safe at doses lower than 15 mg/kg (Tedesco and Haragsim, 2012). Because of its hydrophobicity, it can penetrate cell membranes by an entropy-driven process. Furthermore, it is electrically neutral, which minimizes its toxicity to normal cells (Rezai et al., 2006).

Therefore, in this study we aimed to prepare a CsA-conjugated system for the safe and effective delivery of nanoparticles to cancer cells. To our knowledge, there have been no reports regarding the use of Bor and IR-820 combination treatment for pancreatic cancer. Our study is, therefore, the first of its kind. The developed nanosystem provides a potential avenue for the use of a safe and stable carrier system for effectively penetrating cancer cells. To achieve this, we designed an IR-820-loaded MSN covered with a Bor-loaded PEGylated lipid bilayer modified with CsA (CLMSN/BIR) and evaluated its *in vitro* and *in vivo* anti-cancer effects in pancreatic cancer.

## Experimental section

### Material

Bor was procured from LC Laboratories (Woburn, MA). IR-820, tetraethyl orthosilicate (TEOS), cetyltrimethylammonium bromide (CTAB), ammonium fluoride (NH_4_F), coumarin-6, cholesterol (CHO), and CsA were purchased from Sigma-Aldrich (St Louis, MO). 1,2-Dipalmitoyl-*sn*-glycero-3-phosphocholine (DPPC) and 1,2-distearoyl-*sn*-glycero-3 phosphoethanolamine-*N*-[carboxy(polyethylene-glycol)-2000] (DSPE-PEG) were obtained from NOF America Corporation (White Plains, NY) and Avanti Polar Lipid (Alabaster, AL), respectively. LysoTracker red was purchased from Invitrogen/Molecular Probes, Inc. (Eugene, OR). All other chemicals were of reagent grade and used without further purification.

### Preparation of CsA-amine

CsA-amine is required for the synthesis of DSPE-PEG-CsA. Therefore, a two-step reaction was carried out as described previously (Lu et al., 2001). A schematic representation of the preparation method is provided in Supplementary Figure S1. First, CsA was epoxidized, then it was reacted with an excess of ethylenediamine in tetrahydrofuran for 24 h. Compound III (a by-product) was isolated from solid residue using silica gel chromatography and compound IV (CsA-amine) was obtained as a yellowish solid. ^1^H-NMR and ESI mass spectrometry were performed to confirm the synthesis of CsA-amine.

### Preparation of DSPE-PEG-CsA

CsA-amine was conjugated to DSPE-PEG_2000_-NHS by reacting 26 mg CsA-amine, 58 mg DSPE-PEG_2000_-NHS, and 10 µL trimethylamine in 0.5 mL of dimethylformamide in an argon gas atmosphere under moderate stirring at room temperature (Supplementary Figure S1). The reaction was continued for 3 days and the resulting mixture was dialyzed against deionized water for 48 h to remove impurities. The solution was lyophilized and the resulting crystalline product was stored at −20 °C for further use. Characterization of DSPE-PEG-CsA was performed using MALDI-TOF mass spectrometry (Bruker Daltonics Inc., Fremont, CA).

### Preparation of CLMSN/BIR

**Figure 1.**
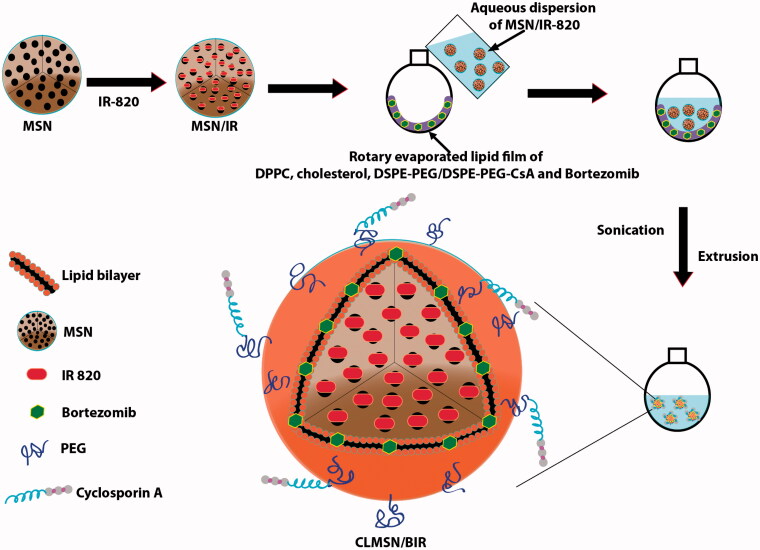
Schematic illustration for the preparation method of CLMSN/BIR.

The schematic representation of the preparation method for CLMSN/BIR is presented in Figure 1. First, MSNs were prepared by dissolving CTAB (213 mg) in distilled water (200 mL) at 80 °C, then NH_4_F (30 mg) was added. This mixture was continuously stirred (1200 rpm) at 80 °C for 1 h. Thereafter, TEOS (1.5 mL) was added dropwise and the mixture was continuously stirred for 2 h until a semi-transparent colloidal state was achieved. High speed centrifugation followed by washing with distilled water resulted in uniformly sized MSNs. IR-820 was dissolved in ethanol and added to the MSN dispersion. The mixture was stirred for 12 h and free IR-820 was removed by dialysis against distilled water. Separately, Bor, DPPC, CHO, and DSPE-PEG/DSPE-PEG-CsA were dissolved in a mixture of chloroform and methanol (4:1) and rotary evaporated for 3 h. An aqueous dispersion of IR-820-loaded MSN was then added to the thin film formed by rotary evaporation. The solution was mixed, then sonicated to develop MSN-loaded vesicles that were then extruded through membranes to obtain CLMSN/BIR. Separately, DSPE-PEG only was used to prepare LMSN/BIR for comparison with CLMSN/BIR.

### Analysis of particle size and zeta potential

The hydrodynamic size, polydispersity index (PDI), and zeta potential of the prepared nanoparticles was analyzed using a Zetasizer Nano ZS (Malvern Instruments, Worcestershire, UK). Three individual measurements were taken for each sample and an average was calculated.

### Spectroscopic evaluations

Ultraviolet (UV)/visible spectra were analyzed to evaluate IR-820 content by conducting measurements at ʎ_max_ = 810 nm using a UV/vis spectrophotometer (PerkinElmer U-2800, Waltham, MA). Fourier-transform infrared (FTIR) spectroscopy analyzes were performed using a Thermo Scientific Nicolet Nexus 670 FTIR spectrophotometer (Thermo Scientific, Waltham, MA).

### Morphological characterization

Morphological characterization of CLMSN/BIR was performed using a transmission electron microscope (TEM, H7600, Hitachi, Tokyo, Japan). A drop of CLMSN/BIR was added onto a carbon-coated copper grid and allowed to adhere after drying. A 2% phosphotungstic acid solution was added for negative staining. The nanoparticle loaded grid was then observed under the TEM.

### Evaluation of photothermal effects

The temperature elevations caused by IR-820 after irradiation with an 808 nm NIR laser (3.0 W/cm^2^) for up to 5 min were evaluated. Different concentrations of IR-820 were used to compare the concentration-dependent photothermal effects of free IR-820 and IR-820-loaded in CLMSN. Changes in temperature were evaluated using a thermal camera (Therm-App TH, Karmiel, Israel).

### *In vitro* drug release study

The Bor release profiles from CLMSN/BIR were determined in phosphate-buffered saline (PBS, pH 7.4) and acetate-buffered saline (ABS, pH 5.0) using a dialysis membrane (Spectra/Por, MWCO 3500 Da, Rancho Dominguez, CA). The tubes containing samples were maintained at 37 °C and shaken at 100 rpm, then 0.5 mL of the release medium was sampled at pre-determined time points. An HPLC analysis of Bor was performed using our previously described method (Thapa et al., 2017) to determine the cumulative drug release pattern. Additionally, NIR on/off cycle treatments were applied to determine the effect of NIR irradiation on the Bor release pattern.

### *In vitro* cell lines and culture

Pancreatic cancer cell lines (PANC-1 and MIA PaCa-2) were obtained from the Korean Cell Line Bank (Seoul, South Korea). These cells were cultured in Dulbecco’s modified Eagle’s medium (DMEM) supplemented with 10% fetal bovine serum (FBS) and 1% penicillin–streptomycin (Hyclone Laboratories, Logan, UT).

### *In vitro* cell cytotoxicity assay

The *in vitro* viabilities of PANC-1 and MIA PaCa-2 cells after treatment with Bor, IR-820, the combination of Bor and IR-820 (BIR), LMSN/BIR, and CLMSN/BIR were evaluated using a 3-(4,5-dimethylthiazol-2-yl)-5-(3-carboxymethoxyphenyl)-2-(4-sulfophenyl)-2H-tetrazolium (MTS) assay (Promega, Madison, WI). Briefly, PANC-1 and MIA PaCa-2 cells were seeded in 96-well plates at a density of 1 × 10^4^ cells per well and incubated for 24 h. Cells were then treated with different concentrations of Bor, IR-820, BIR, LMSN/BIR, and CLMSN/BIR and further incubated for 24 h. Untreated cells served as controls. The cells were treated with MTS solution and the absorbance was measured at 493 nm using an automated microplate reader.

### Cellular uptake study

*In vitro* cellular uptake of coumarin-6-loaded CLMSN in PANC-1 and MIA PaCa-2 cells was qualitatively studied using confocal microscopy. The cancer cells were seeded onto coverslips, placed in 12-well plates, incubated overnight, and treated with coumarin-6-loaded CLMSN for 1 h. LysoTracker Red was then added and the cells were further incubated for 10 min. Thereafter, the media were removed and the cells were washed twice with PBS, then fixed with 4% paraformaldehyde for 10 min in the dark. Finally, cells were washed with PBS, mounted on glass slides, and sealed to capture images using a confocal laser scanning microscope (CLSM, Leica Microsystems, Wetzlar, Germany).

Quantitative cellular uptake of CLMSN in pancreatic cancer cells was determined using flow cytometry. Coumarin-6 was used as a fluorescence probe. PANC-1 and MIA PaCa-2 cells were grown in 12-well plates and incubated with coumarin-6-loaded CLMSN at different concentrations (0.5, 1.0, and 2.0 µg/mL) and for different amounts of time (30, 60, and 90 min). Cells were then harvested and washed with PBS and the intracellular fluorescence was measured using a fluorescence-activated cell sorting (FACS) Verse flow cytometer (BD Biosciences, San Jose, CA).

### Intracellular ROS detection

PANC-1 and MIA PaCa-2 cells were seeded in 12-well plates and incubated for 24 h. The cells were then washed with PBS and treated with freshly prepared DCFH-DA solution for 1 h. Finally, the media containing dye solution were removed and cells were washed with PBS, then treated for 6 h with Bor, IR-820, BIR, LMSN/BIR, or CLMSN/BIR with or without NIR irradiation. The harvested cells were subsequently analyzed using FACS.

### Live/dead assay

PANC-1 and MIA PaCa-2 cells were seeded in 12-well plates and incubated for 24 h. Thereafter, the cells were treated with Bor, IR-820, BIR, LMSN/BIR, or CLMSN/BIR. For the NIR treatment groups, after incubation with free drugs or formulations for 6 h, an 808 nm NIR laser with a power output of 3.0 W/cm^2^ was used to irradiate the cells for 5 min. Subsequently, the cells were stained with acridine orange (AO) and propidium iodide (PI) in PBS at final concentrations of 6.7 and 750 µM, respectively, and photographed using a fluorescence microscope (Nikon Eclipse Ti, Nikon Instruments Inc., NY, USA).

### Cellular apoptosis study

The apoptotic effects of Bor, IR-820, BIR, LMSN/BIR, and CLMSN/BIR with or without NIR laser irradiation were analyzed in PANC-1 and MIA PaCa-2 cells using flow cytometry. First, the cells were grown in 12-well plates and treated with the drugs and formulations for 24 h. Thereafter, the cells were harvested, washed with PBS, stained with Annexin-V/PI, and analyzed using an FACS Verse flow cytometer (BD Biosciences).

### Cell migration study

The anti-migration effects of Bor, IR-820, BIR, LMSN/BIR, and CLMSN/BIR treatments in PANC-1 and MIA PaCa-2 cells were analyzed using Transwell assays as described in our previous study (Thapa et al., 2016).

### *In vivo* imaging and photothermal effects

All animal experiments were conducted in compliance with the relevant laws and institutional guidelines and approved by the local ethics committee. Cy5.5-loaded CLMSN were used to investigate tumor targeting efficacy. Female BALB/c nude mice bearing PANC-1 tumor xenografts were established by subcutaneous injection of 2 × 10^7^ cells/100 µL into the right thigh flanks (Orient Ltd., Seoul, South Korea). Imaging was performed when the tumor volume reached ∼500 mm^3^. Following intravenous injection of drug formulations via the tail veins, their distribution was observed after 24 h using an *in viv*o imaging apparatus (FOBI, NeoScience Co., Ltd, Seoul, South Korea). Mice were sacrificed after 24 h and the tumor tissues and organs were excised. *Ex vivo* fluorescence images of the tumor, heart, lung, spleen, kidney, and liver were obtained.

The *in vivo* photothermal effect of intravenously administered CLMSN/BIR was evaluated using digital photographs taken with a thermal camera (Therm-App TH). Briefly, the tumors of mice treated with intravenous injections of CLMSN/BIR were irradiated with an 808 nm NIR laser (3.0 W/cm^2^) for up to 5 min. Digital images of the irradiated tumors were taken to determine the induction of photothermal effects.

### *In vivo* antitumor study

Xenograft mice models bearing PANC-1 cells were established by subcutaneous injection of 2 × 10^7^ cells/100 µL into the right thigh flanks of 7-week old female BALB/c nude mice (Orient Ltd., Pusan, South Korea). Once the tumor volumes reached 100 mm^3^, animals were randomly divided into seven groups (*n* = 6 per group). The mice were injected with a combination of Bor and IR-820, LMSN/BIR, or CLMSN/BIR via the tail vein four times every 3 days. The day following the injection, the NIR treatment groups were irradiated with NIR lasers (808 nm, 3.0 W/cm^2^, 5 min). Tumor sizes (length and width) in each group were measured using a Vernier caliper and the change in tumor volume was determined using the following formula: $Volume=\frac{\left(Length\times Width^{2}\right)}{2.}$ Changes in the body weight of the mice were also calculated to assess for possible toxicity. The animals were sacrificed after four weeks of treatment and the primary tumors along with the organs (heart, liver, lung, kidney, and spleen) were surgically excised for immunohistochemical analysis.

### Histopathological and immunohistochemical analysis

The excised tumor masses were cut and fixed in 10% neutral buffered formalin, embedded in paraffin, serially sectioned (3–4 µm), and stained with hematoxylin and eosin (H&E) for optical microscopy (Nikkon Corp., Tokyo, Japan). The tumor cell volumes and intact tumor cell-occupied regions (%/mm^2^ of the tumor mass) were calculated using an automated computer-based image analyzer (*i*Solution FL ver 9.1; IMT *i*-solution Inc., Vancouver, Quebec, Canada). Expression of caspase-3, poly[adenosine diphosphate (ADP)-ribose] polymerase (PARP), cluster of differentiation 31 (CD31), and Ki-67 was evaluated immunohistochemically using purified primary and biotinylated secondary antibodies with avidin-biotin-peroxidase complex (ABC) and a peroxidase substrate kit (Vector Labs, Burlingame, CA). The tumor samples were incubated with primary antisera overnight at 4 °C, then treated with a biotinylated secondary antibody and ABC reagents for 1 h. Thereafter, the total area covered for each marker was determined. The marker expression was considered positive if >20% of the cells were covered as calculated using an automated image analyzer (%/mm^2^ of tumor mass).

### Statistical analysis

All the experimental results are expressed as mean ± standard deviation (SD). Student’s *t*-test for pairs of groups and one-way analysis of variance for multiple groups were used for to determine statistical significance among the groups. The data were considered statistically significant at *p* < .05.

## Results and discussion

### Preparation and characterization of CLMSN/BIR

The HBP, CsA, was conjugated with the nanosystem to assist with the transport of nanoparticles into the cancer cells. Therefore, CsA-amine was synthesized and the prepared samples were identified using ^1^H-NMR spectroscopy (Supplementary Figures S1, S2a, and S2b). In addition to the characteristic ^1^H-NMR spectra of CsA, additional spectra of CsA-amine (δ, ppm, DMF-d_7_) at 8.25 (m, 4H, CONH) and 2.99 (m, 21 H, NCH_3_) (Lu et al., 2001) were observed that indicated the successful synthesis of CsA-amine. Further confirmation was performed using ESI mass spectrometry (Supplementary Figure S2c), wherein CsA-amine exhibited m/z: 639 [(M^+^+2)/2], 1278 (M^+^+1), as observed in a previous report (Lu et al., 2001). This CsA-amine was then conjugated with DSPE-PEG-NHS to synthesize DSPE-PEG-CsA conjugate. This conjugate synthesis was confirmed using MALDI-TOF mass spectrometry (Supplementary Figure S3), wherein DSPE-PEG-NHS exhibited spectra for DSPE at m/z ∼750 and for PEG at m/z ∼2000, corresponding to their molecular weights. DSPE-PEG-CsA presented an additional spectrum at m/z ∼1300, corresponding to the molecular weight of CsA-amine conjugated to the DSPE-PEG-NHS. A lipid bilayer was then prepared using the DSPE-PEG-CsA.

**Figure 2.**
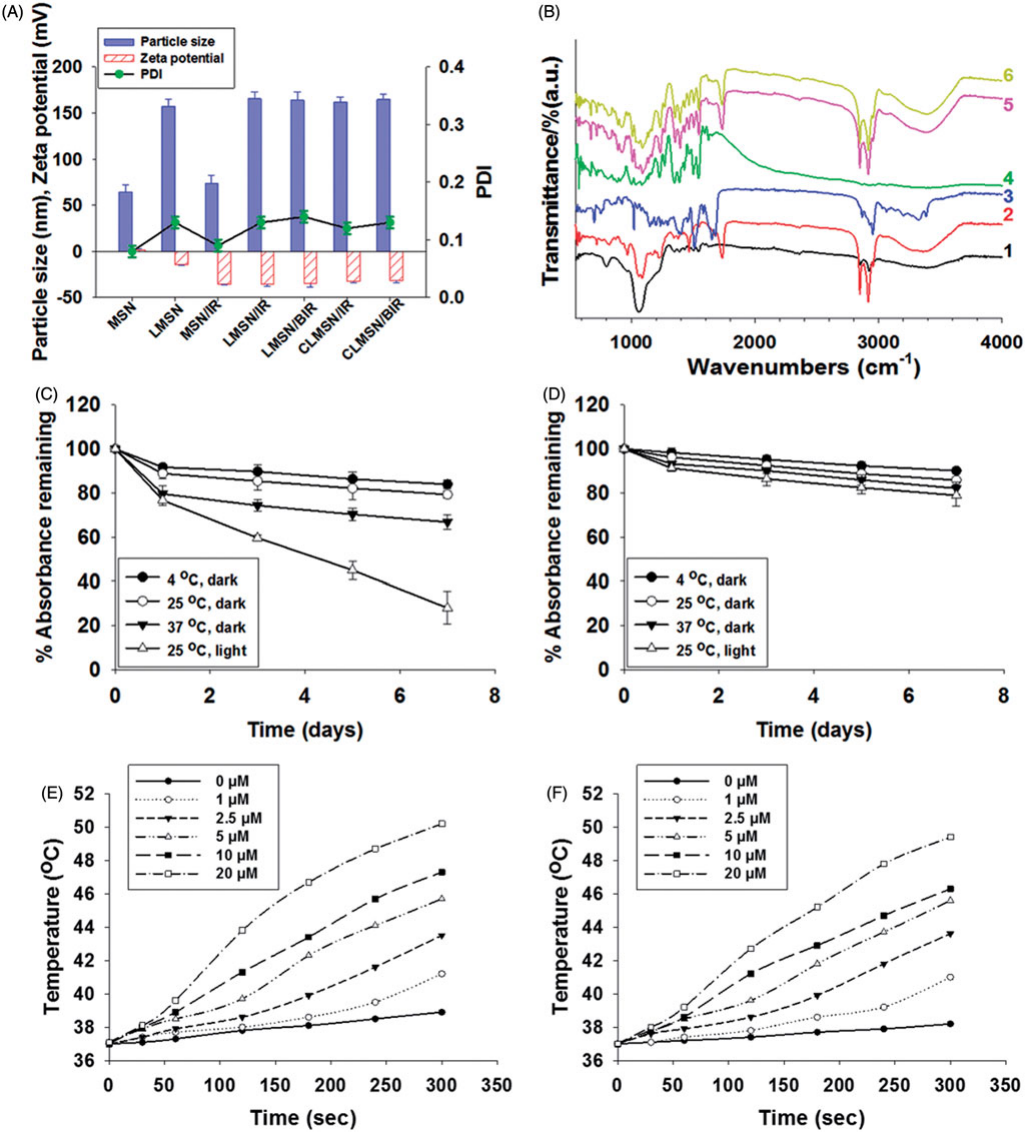
(A) Particle size, PDI, and zeta potential of blank and drug-loaded CLMSNs. (B) FTIR spectra for (1) blank MSN, (2) blank LMSN, (3) Bor, (4) IR-820, (5) LMSN/BIR, and (6) CLMSN/BIR. Stability of IR-820 in the (C) free- and (D) CLMSN-loaded forms under different temperature and light/dark conditions. Temperature elevations caused by IR-820 in (E) free- and (F) CLMSN-loaded forms at different concentrations following NIR irradiation (808 nm, 3.0 W/cm^2^) for up to 5 min.

The preparation of CLMSN/BIR included a hybrid combination of MSN and a lipid bilayer structure (Figure 1). Therefore, MSNs were separately prepared using CTAB, NH_4_F, and TEOS with an average particle size of ∼70 nm and slightly positive zeta potential (Figure 2(A)). The MSNs were then coated with a lipid bilayer using a thin film hydration technique to form LMSN or CLMSN. The composition of the lipid bilayer included DPPC, CHO, and DSPE-PEG/DSPE-PEG-CsA. A 1:1 ratio of DSPE-PEG and DSPE-PEG-CsA was used for the formation of the CsA-conjugated nanosystem. With the addition of the lipid bilayer, the nanoparticle size increased to ∼160 nm.

IR-820 was successfully loaded onto the MSNs with a loading capacity of 10.0 ± 0.8%. IR-820 is an amphiphilic dye that possesses both hydrophilic and hydrophobic properties, which allow it to assist in the hydrophobic–hydrophobic interaction with MSNs for effective loading. Bor is a hydrophobic drug that can easily accommodate itself within the lipid bilayer system, resulting in high drug-loading capacity (Thapa et al., 2017). The incorporation of both Bor and IR-820 within the LMSN did not increase the final particle size. Furthermore, CsA conjugation did not influence the particle size of CLMSN/BIR and its size remained ∼160 nm (Figure 2(A)). FTIR analysis indicated the characteristic peaks of MSN at 1050 and 3400 cm^−1^, whereas lipid bilayer-wrapped MSN presented peaks at 1050, 1400, 1800, 2800, 3000, and 3400 cm^−1^, corresponding to the components of the lipid bilayer (Figure 2(B)). Bor and IR-820 exhibited characteristic peaks below 2000 cm^−1^. The final formulation, CLMSN/BIR, presented the characteristic peaks of the lipid bilayer, thereby confirming the successful incorporation of IR-820-loaded MSN within the lipid bilayer and the successful incorporation of Bor.

**Figure 3.**
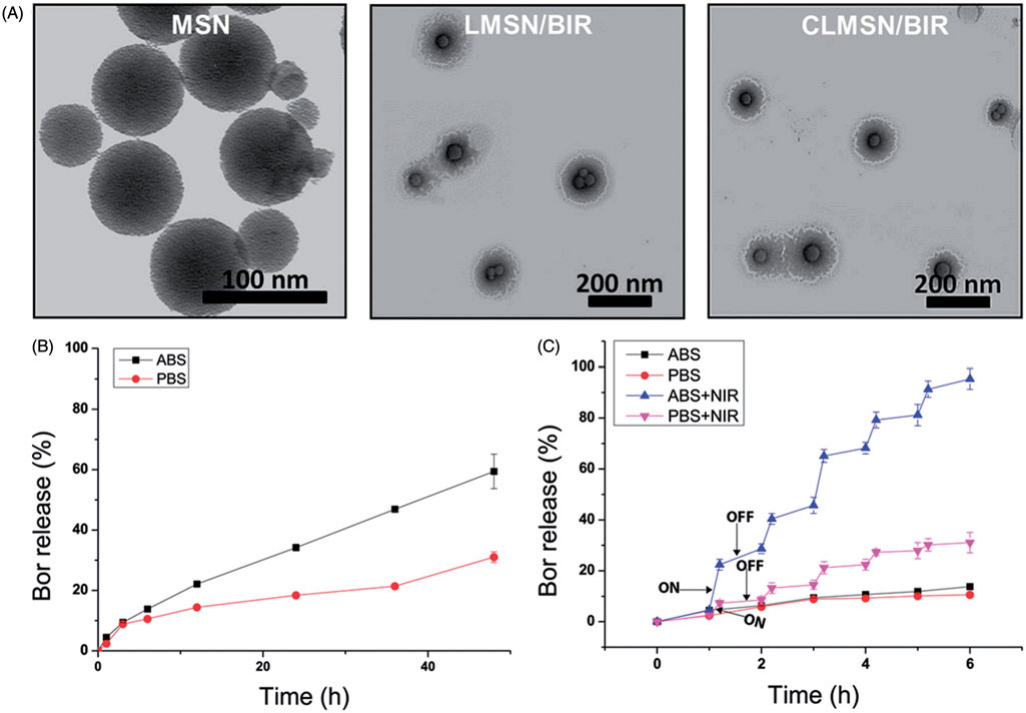
(A) TEM images of MSN, LMSN/BIR, and CLMSN/BIR. *In vitro* drug release profiles of Bor from CLMSN/BIR in (B) PBS and ABS and (C) with or without NIR irradiation (808 nm, 3.0 W/cm^2^). NIR irradiation was performed for 5 min with on/off cycles on alternating hours.

The stability of IR-820, in solution form and MSN-loaded form, was evaluated under light and dark conditions and at different temperatures (Figure 2(C,D)). The results indicated that light was a major source of degradation for IR-820. Although small, a temperature-dependent degradation of IR-820 was also evident. These results were consistent with those observed by Fernandez-Fernandez et al. (2014). The loading of IR-820 in the MSNs improved their stability under light and temperature conditions, which was attributed to the ability of MSN to hold IR-820 within the nanoparticles for their protection (Lei et al., 2014). In addition, the photothermal effects of free IR-820 and MSN-loaded IR-820 were compared (Figure 2(E,F)). A concentration-dependent photothermal effect was observed for free IR-820 after irradiation with an NIR laser at 808 nm (3.0 W/cm^2^). Even with the incorporation in MSNs, there was little change in the pattern of the photothermal effects of IR-820, suggesting our nanosystem was useful for causing photothermal ablation effects. Morphological characterization of MSN, LMSN/BIR, and CLMSN/BIR was performed using TEM (Figure 3(A)). MSNs of uniform size were prepared and a uniform lipid bilayer coating was evident, suggesting successful synthesis of a hybrid lipid-MSN. The stability of the prepared LMSN/BIR and CLMSN/BIR was evaluated in different media (water, PBS, ABS, and DMEM) for up to 48 h (Supplementary Figure S4). Both nanoparticles presented similar patterns of changes in particle size, PDI, and zeta potential in different media. Higher stability was observed in water and PBS and particle size increased slightly in DMEM. Under acidic conditions in ABS, particle size drastically increased with changes in zeta potential and PDI for both LMSN/BIR and CLMSN/BIR. Morphological analysis of CLMSN/BIR in different media was further performed (Supplementary Figure S5) and the results agreed with those from the DLS measurements. Following NIR irradiation, the lipid bilayer was highly disrupted for nanoparticles dispersed in ABS rather than PBS.

### *In vitro* drug release profiles

The *in vitro* drug release profiles for Bor were determined in PBS and ABS for both LMSN/BIR and CLMSN/BIR (Figure 3(B) and Supplementary Figure S6). Higher Bor release was observed in acidic conditions (pH 5.0) than in normal physiological conditions (pH 7.4), which is highly beneficial for tumor-targeted release (Thapa et al., 2015). Furthermore, drug release patterns following 808 nm NIR laser irradiation were determined (Figure 3(C)). With the ‘ON’ cycle for the NIR laser, there was a pulsed increase in the release of Bor in PBS and an abrupt increase in Bor release in ABS was evident. Lower stability of the lipid bilayer system in acidic conditions, followed by temperature elevation induced disruption, might have contributed to abrupt Bor release in acidic pH conditions (Ramasamy et al., 2014).

### *In vitro* cellular uptake studies

**Figure 4.**
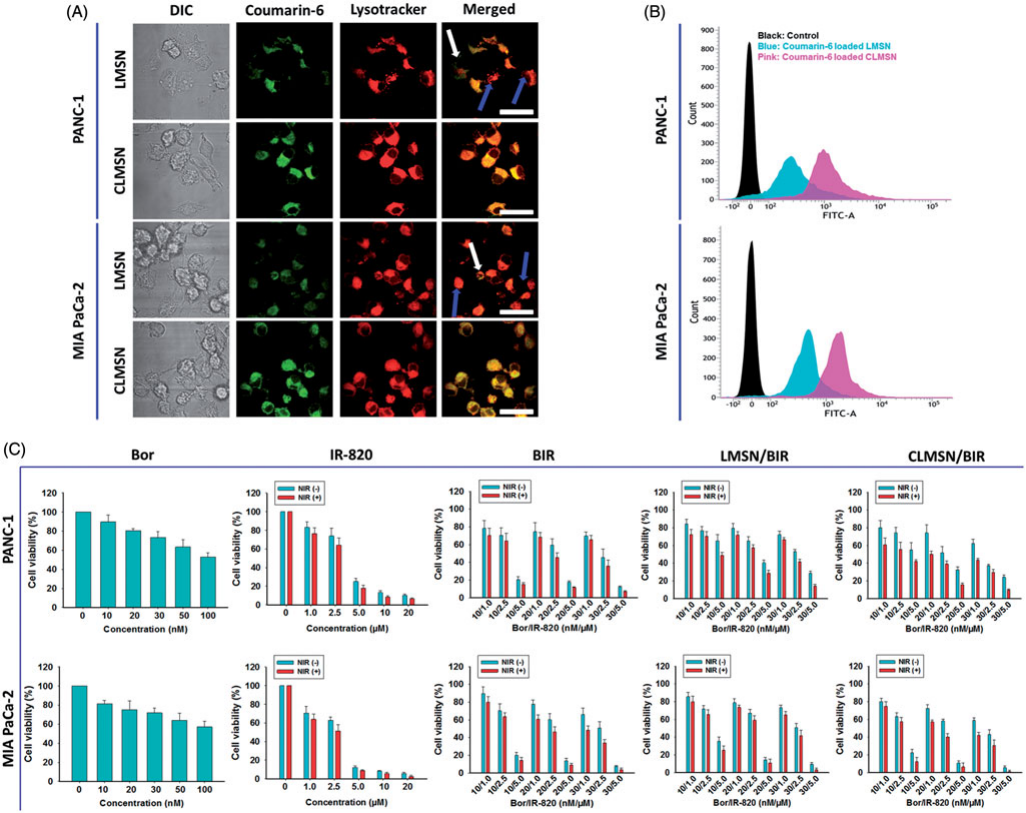
(A) Confocal images of the cellular uptake of coumarin-6-loaded LMSN and CLMSN in PANC-1 and MIA PaCa-2 cells (white arrows: non-lysosomal uptake, blue arrows: minimal lysosomal uptake; scale bar: 20 µm). (B) FACS analysis of the cellular uptake of coumarin-6-loaded LMSN and CLMSN in PANC-1 and MIA PaCa-2 cells. (C) *In vitro* cytotoxicity of Bor, IR-820, BIR, LMSN/BIR, and CLMSN/BIR in PANC-1 and MIA PaCa-2 cells after 24 h of treatment in the presence or absence of NIR irradiation (808 nm, 3.0 W/cm^2^, 5 min). Please check the online version of colored figures for details.

Comparative cellular uptake studies of coumarin-6-loaded LMSN and CLMSN were performed using confocal imaging and FACS analysis (Figure 4). Confocal images indicated that coumarin-6-loaded LMSN was minimally taken up in both PANC-1 and MIA PaCa-2 cells. However, coumarin-6-loaded CLMSN was highly taken up and the nanoparticles were concentrated in lysosomes, which could be highly beneficial for the targeted release of drugs inside the cancer cells (Figure 4(A)). These results were quantitatively supported by FACS. CLMSN was taken up in a concentration- and time-dependent manner in both PANC-1 and MIA PaCa-2 cells (Supplementary Figure S7). Additionally, a comparative analysis indicated a greater uptake of CLMSN than of LMSN in both pancreatic cancer cell lines (Figure 4(B)). These results can be attributed to the presence of conjugated CsA in CLMSN, which acted as a driving force for nanoparticle delivery into the cancer cells (Gao et al., 2016).

### Cell viability studies

The *in vitro* cell cytotoxicity studies of PANC-1 and MIA PaCa-2 cells after treatment with Bor, IR-820, BIR, LMSN/BIR, and CLMSN/BIR with or without NIR treatment are presented in Figure 4(C). Different ratios of Bor and IR-820 were used to determine their synergistic combination effects. Free drug combinations of BIR at 10/2.5 and 20/2.5 (nM/µM) were highly effective for inducing cytotoxicity. At these ratios, compared to those of LMSN/BIR, the cytotoxic effects induced by CLMSN/BIR were pronounced. The hydrophobic interaction of CsA with cell membranes (Rezai et al., 2006) aided in the lysosomal uptake of the nanoparticles, which then released Bor for anticancer effects. NIR irradiation of IR-820 resulted in the photothermal ablation of cancer cells.

### *In vitro* ROS generation

**Figure 5.**
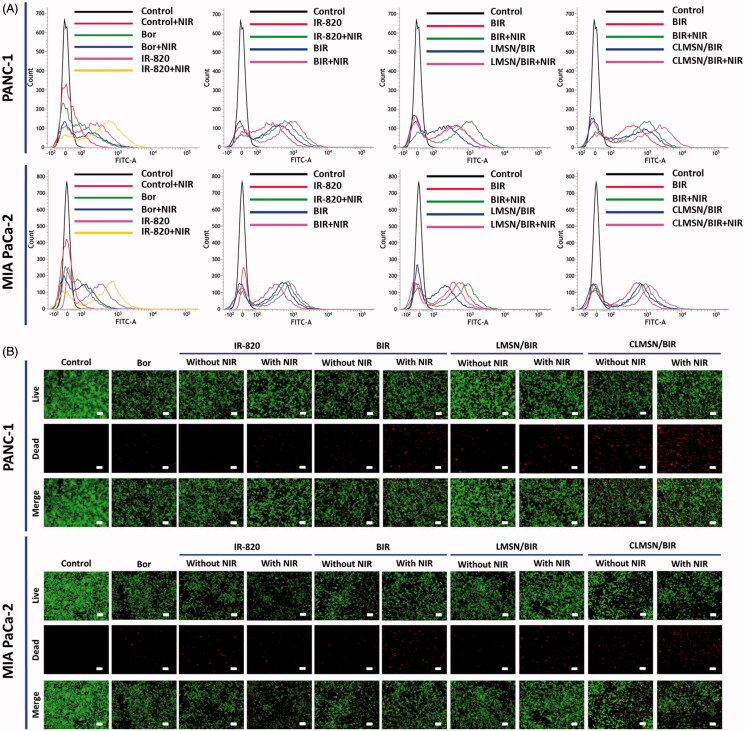
(A) FACS analyzes for determination of intracellular ROS production and (B) live/dead staining assay for determination of the effect of Bor, IR-820, BIR, LMSN/BIR, and CLMSN/BIR treatments on PANC-1 and MIA PaCa-2 cells with or without NIR (808 nm, 3.0 W/cm^2^, 5 min) laser exposure. Live cells were stained green with acridine orange and dead cells were stained red with propidium iodide; scale bar: 50 µm. Please check the online version of colored figures for details.

One of the important mechanisms of the anticancer effect of IR-820 is mediated by the ROS generation that occurs after irradiation with an NIR laser (Zhou et al., 2016). Therefore, ROS generation in PANC-1 and MIA PaCa-2 cells was studied after treatment with Bor, IR-820, BIR, LMSN/BIR, and CLMSN/BIR with or without NIR laser irradiation (Figure 5(A)). As expected, the NIR laser irradiation of IR-820 resulted in increased ROS generation compared to that observed without NIR. Combining BIR with NIR further enhanced ROS generation, which suggested a synergistic effect of the combination for the induction of cancer cell apoptosis. Combining LMSN/BIR with NIR did not enhance ROS generation owing to low uptake; however, combining CLMSN/BIR with NIR greatly enhanced ROS generation, which could contribute greatly to the induction of apoptosis of pancreatic cancer cells. The ROS produced are highly reactive with biological molecules and could cause irreversible oxidative damage and cell death (Trachootham et al., 2009).

### Cellular apoptosis studies

A live/dead assay was used to qualitatively evaluate chemo-phototherapy treatment in pancreatic cancer cells (Figure 5(B)). Green color represented live cells, whereas red represented dead cells. For all the treatments, NIR laser irradiation enhanced the number of dead cells in both PANC-1 and MIA PaCa-2 cells. CLMSN/BIR treatment along with NIR laser irradiation caused an increase in cancer cell apoptosis, suggesting the effects of Bor and IR-820 were synergistic and mediated by the hydrophobic driving force of CsA.

**Figure 6.**
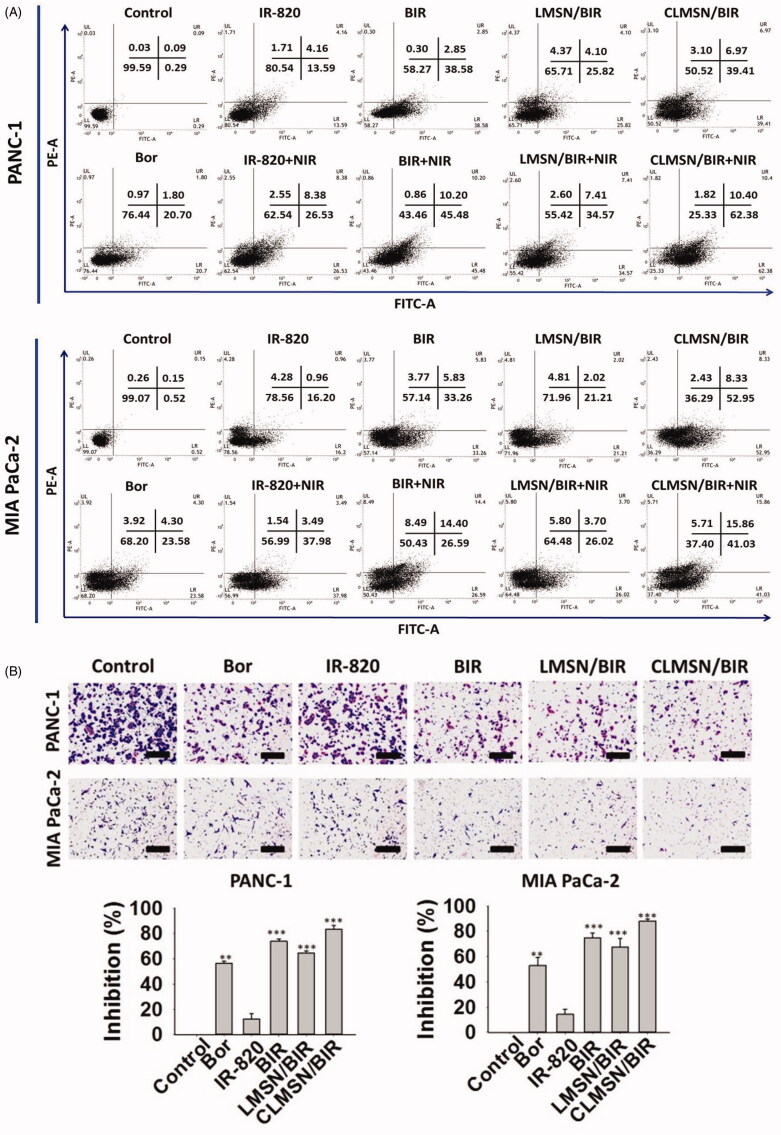
Determination of (A) cellular apoptosis and (B) cell migration of PANC-1 and MIA PaCa-2 cells (scale bar: 100 µm). The cells were treated with Bor, IR-820, BIR, LMSN/BIR, or CLMSN/BIR in the presence or absence of NIR exposure (808 nm, 3.0 W/cm^2^, 5 min).

Quantitative cellular apoptosis was determined using FACS analysis (Figure 6(A)). Compared to either agent alone (Bor or IR-820), treatment with the combination resulted in enhanced apoptotic effects in both PANC-1 and MIA PaCa-2 cells. This effect was further improved with NIR laser irradiation of the treated cells. The lack of interaction between LMSN/BIR and the cell membrane might have resulted in lower uptake and, therefore, the apoptotic effects were limited. However, treatment with CLMSN/BIR resulted in greatly enhanced apoptosis after NIR irradiation of the treated cells because of the hydrophobic interaction between the conjugated CsA and the cell membranes, which resulted in improved cellular uptake of the nanoparticles.

### Cell migration studies

Epithelial to mesenchymal transition (EMT) results in the metastasis of cancer cells. Pancreatic cancer cells possess the ability to migrate to distant organs (Zhou et al., 2017). Therefore, cell migration studies were carried out and the anti-migration effects of the free drugs, the drug combination, and the final formulation were evaluated (Figure 6(B)). As observed, Bor was effective for reducing cell migration; however, IR-820 was not. The combination of Bor and IR-820 further improved the anti-migration effects. Importantly, the anti-migration effects of CLMSN/BIR were significantly better than those of LMSN/BIR, thereby indicating that the HBP and hybrid lipid-MSN system were effective for controlled and targeted drug release.

### *In vivo* imaging and photothermal effects

**Figure 7.**
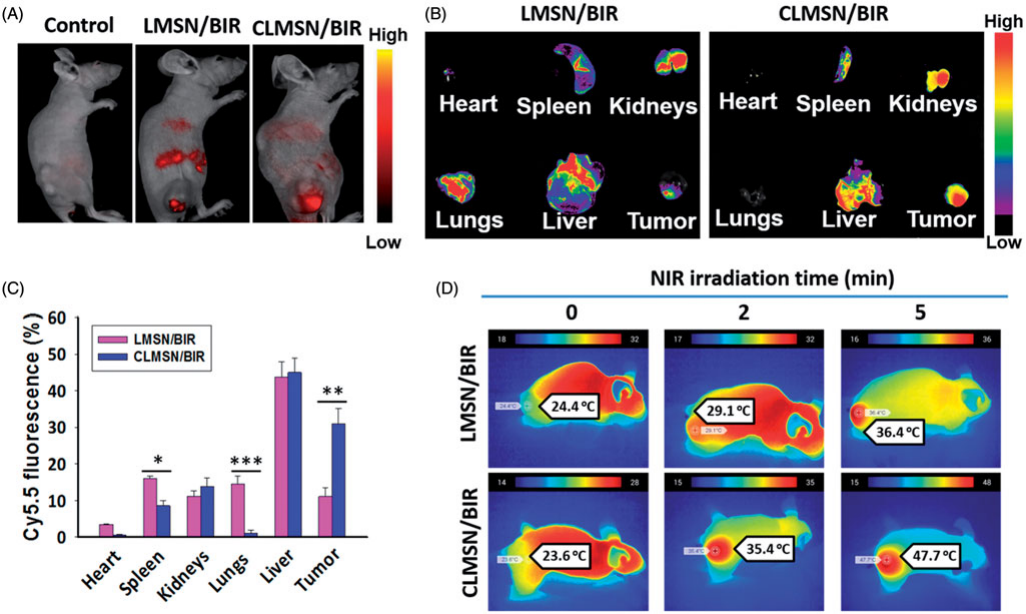
(A) *In vivo* biodistribution of Cy5.5-loaded LMSN and CLMSN in PANC-1 tumor xenograft mice. (B) Imaging and (C) evaluation of Cy5.5-loaded LMSN and CLMSN distribution in different organs after intravenous administration (*p < .05; **p < .01; ***p < .001). (D) *In vivo* photothermal imaging of NIR laser irradiated tumors pretreated with CLMSN/BIR formulation.

The *in vivo* biodistribution of Cy5.5-loaded LMSN and CLMSN nanoparticles was evaluated using an *in vivo* imaging system. After a 24-h intravenous administration of Cy5.5-loaded LMSN and CLMSN nanoparticles, imaging was performed, which indicated greater accumulation of the CLMSN nanoparticles in the tumor area compared to that of the LMSN nanoparticles (Figure 7(A)). Furthermore, *ex vivo* imaging of organs showed the distribution of CLMSN nanoparticles in organs like the kidneys and liver along with high accumulation in the tumor (Figure 7(B,C)). However, a significantly lower distribution in tumor was observed for LMSN nanoparticles, along with a significantly higher accumulation in the spleen and lungs. The tumors of mice administered LMSN/BIR and CLMSN/BIR intravenously were irradiated using an NIR laser to determine the photothermal effects of the formulations (Figure 7(D)). After irradiation for 2 and 5 min, a significant increase in the tumor temperature was clearly visible in the CLMSN/BIR-treated group compared to that in the LMSN/BIR-treated group. This result was attributable to a higher uptake and the subsequent photothermal effects of the incorporated IR-820 in the CLMSN/BIR formulation.

### *In vivo* antitumor efficacy of combination therapy

**Figure 8.**
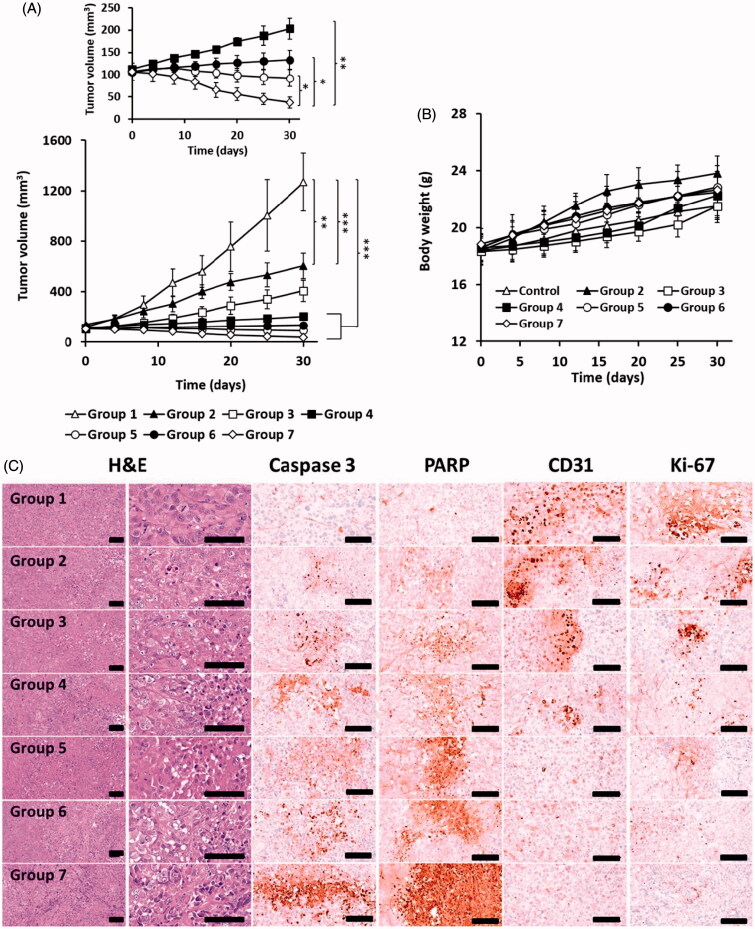
*In vivo* antitumor study: (A) tumor growth and (B) weight gain/loss profiles in PANC-1 xenograft mice after treatment. (C) Immunohistochemical evaluation of PANC-1 xenograft tumors in mice of different treatment groups. Group 1: Control, Group 2: BIR, Group 3: BIR + NIR, Group 4: LMSN/BIR, Group 5: LMSN/BIR + NIR, Group 6: CLMSN/BIR, Group 7: CLMSN/BIR + NIR (NIR exposure: 808 nm, 3.0 W/cm^2^, 5 min). *p < .05, **p < .01, ***p < .001. Changes in the levels of Caspase-3, PARP, CD-31 and Ki-67 expressions in tumor cells of treated groups, scale bars: 120 μm.

The synergistic anticancer effects of the free drugs and formulations were evaluated in PANC-1 xenograft mouse models (Figure 8(A)). Compared to that observed with control, a significant reduction in tumor volume was observed with BIR combination treatment. This effect was further enhanced when the BIR-treated mice tumors were irradiated with NIR, which was attributable to the combination chemo-phototherapy. Use of the LMSN and CLMSN formulations significantly reduced the tumor mass. Nanoparticles can reach the tumor site by EPR effects for cargo delivery of chemotherapeutics, which leads to enhanced anti-tumor effects. Furthermore, the greatest inhibition of tumor growth was observed in CLMSN/BIR + NIR-treated mice. Conjugation of CsA to the nanosystem allowed the formulation to penetrate the cancer cells and the combination of chemo- and phototherapy resulted in the highest anti-cancer effects in xenograft models.

Potential toxic effects of the free drugs and formulations were evaluated by measuring body weight (Figure 8(B)) and performing organ histopathological evaluations (Supplementary Figure S8) in the treated mice. In all the groups, there was no reduction in body weight, suggesting the toxic effects of free drugs and formulations were minimal. Furthermore, the histopathological images of the organs (heart, liver, lung, kidney, and spleen) from all the groups revealed no abnormal findings. These results indicated the potential of the nanoparticle formulations to effectively deliver chemotherapeutics to the cancer cells for chemo-phototherapy.

Further, the antitumor effects were clearly depicted by the caspase-3, PARP, CD31, and Ki-67 levels (Figure 8(C)). The apoptosis markers (caspase-3 and PARP) increased to the highest extent and the proliferation markers (CD31 and Ki-67) decreased to the lowest extent after treatment with CLMSN/BIR + NIR compared to that observed with the other treatments. Compared to LMSN, the conjugation of CsA to the system further improved the anticancer effects of the nanosystem by enhancing the uptake of the nanosystems within the tumors.

## Conclusions

A CsA-conjugated hybrid lipid-MSN system was successfully developed with incorporated Bor and IR-820. The nanosystem had a particle size of ∼160 nm, which was suitable for reaching the tumor site through the EPR effect. These nanoparticles interacted more highly with pancreatic cancer cells mediated by CsA, resulting in high cellular uptake and apoptotic effects caused by combination chemo-phototherapy. Enhanced tumor site accumulation of the nanoparticles was evident, which caused an increase in the photothermal effects upon irradiation of PANC-1 tumor xenografts in mice. Additionally, the potent antitumor effects of CLMSN/BIR were revealed by a significant reduction in tumor volume and an increase in apoptosis markers (caspase-3 and PARP). Therefore, these HBP-conjugated nanosystems could be used for effective chemo-phototherapy of pancreatic cancer.

## Supplementary Material

IDRD_Kim_et_al_Supplemental_Content.docx
