# Supplementary material for: Hydrophobic binding peptide-conjugated hybrid lipid-mesoporous silica nanoparticles for effective chemo-photothermal therapy of pancreatic cancer
Source: Drug Deliv. 2017 Nov 3;24(1):1690–702. doi: 10.1080/10717544.2017.1396382 (PMC8240994; doi:10.1080/10717544.2017.1396382)
Supplement: IDRD_Kim_et_al_Supplemental_Content.docx [file IDRD_A_1396382_SM2224.docx]

**Supplementary Material**

**Hydrophobic Binding Peptide-Conjugated Hybrid Lipid-Mesoporous Silica Nanoparticles for Effective Chemo-Photothermal Therapy of Pancreatic Cancer**

Raj Kumar Thapa^1^, Hanh Thuy Nguyen^1^, Milan Gautam^1^, Aarajana Shrestha^1^, Eung Seok Lee^1^, Sae Kwang Ku^2^, Han-Gon Choi^3^, Chul Soon Yong^1**^, and Jong Oh Kim^1*^

^1^College of Pharmacy, Yeungnam University, 280 Daehak-Ro, Gyeongsan, Gyeongsanbuk-do, 712-749, Republic of Korea

^2^College of Korean Medicine, Daegu Haany University, Gyeongsan, 712-702, South Korea

^3^College of Pharmacy, Hanyang University, 55, Hanyangdaehak-ro, Sangnok-gu, Ansan 426-791, Republic of Korea

**^*^**Corresponding author: Prof. Jong Oh Kim, Ph.D.

Tel: +82-53-810-2813

Fax: +82-53-810-4654

E-mail: jongohkim@yu.ac.kr

^**^ Co-corresponding author: Prof. Chul Soon Yong, Ph.D.

Tel: +82-53-810-2812

Fax: +82-53-810-4654

E-mail: csyong@ynu.ac.kr

**Figure S1.** Schematic representation for the preparation method of DSPE-PEG-CsA; (1) m-chloroperoxybenzoic acid, anhydrous Na_2_CO_3_, methylene chloride, reaction overnight at room temperature; (2) ethylenediamine, tetrahydrofuran, refluxing for 24 h; (3) dimethylformamide, stirring in nitrogen gas atmosphere for 3 days at room temperature.


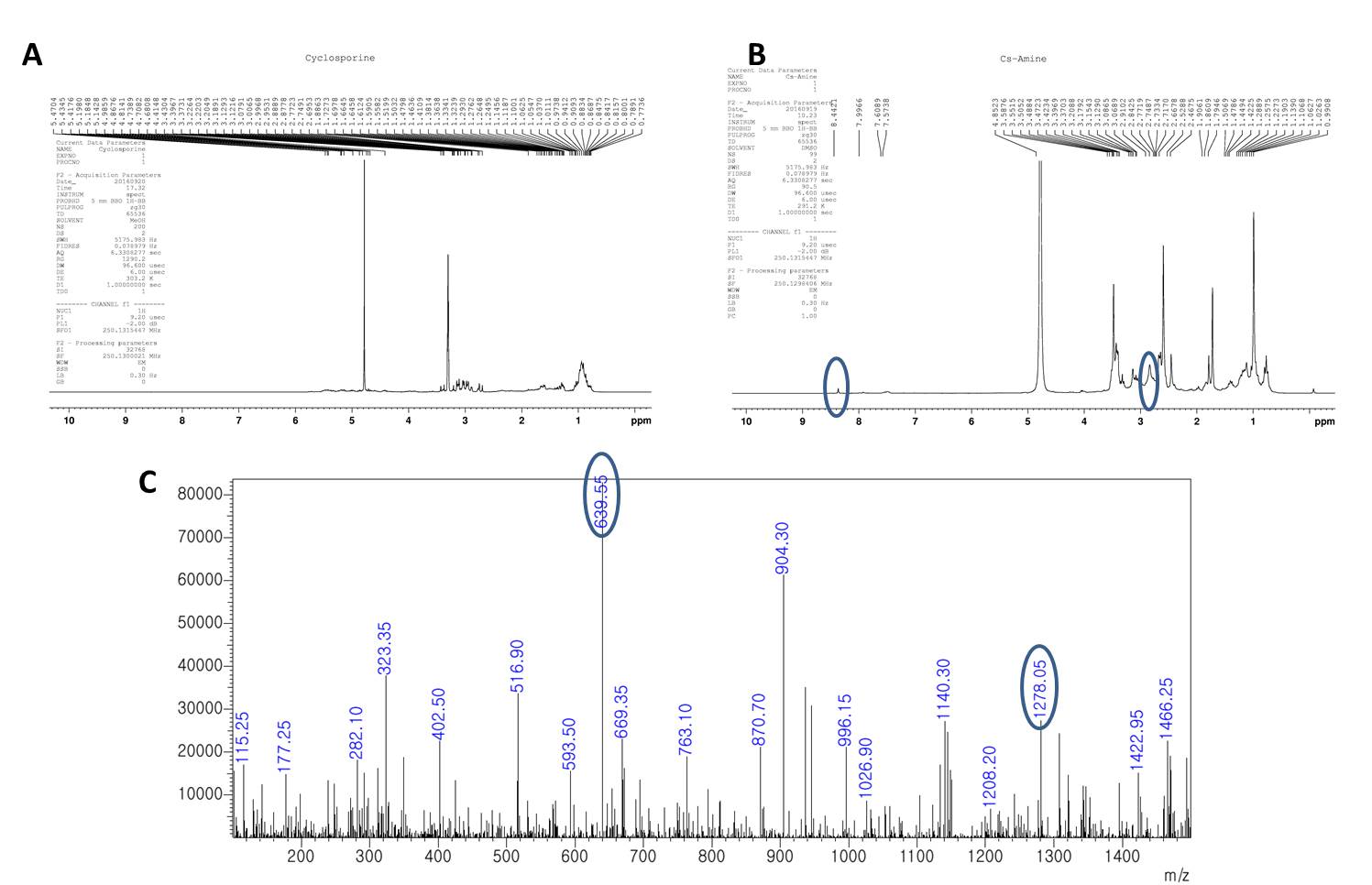


**Figure S2**. ^1^H-NMR spectra for (A) CsA and (B) CsA-amine. (C) Mass spectroscopy analysis of CsA-amine.


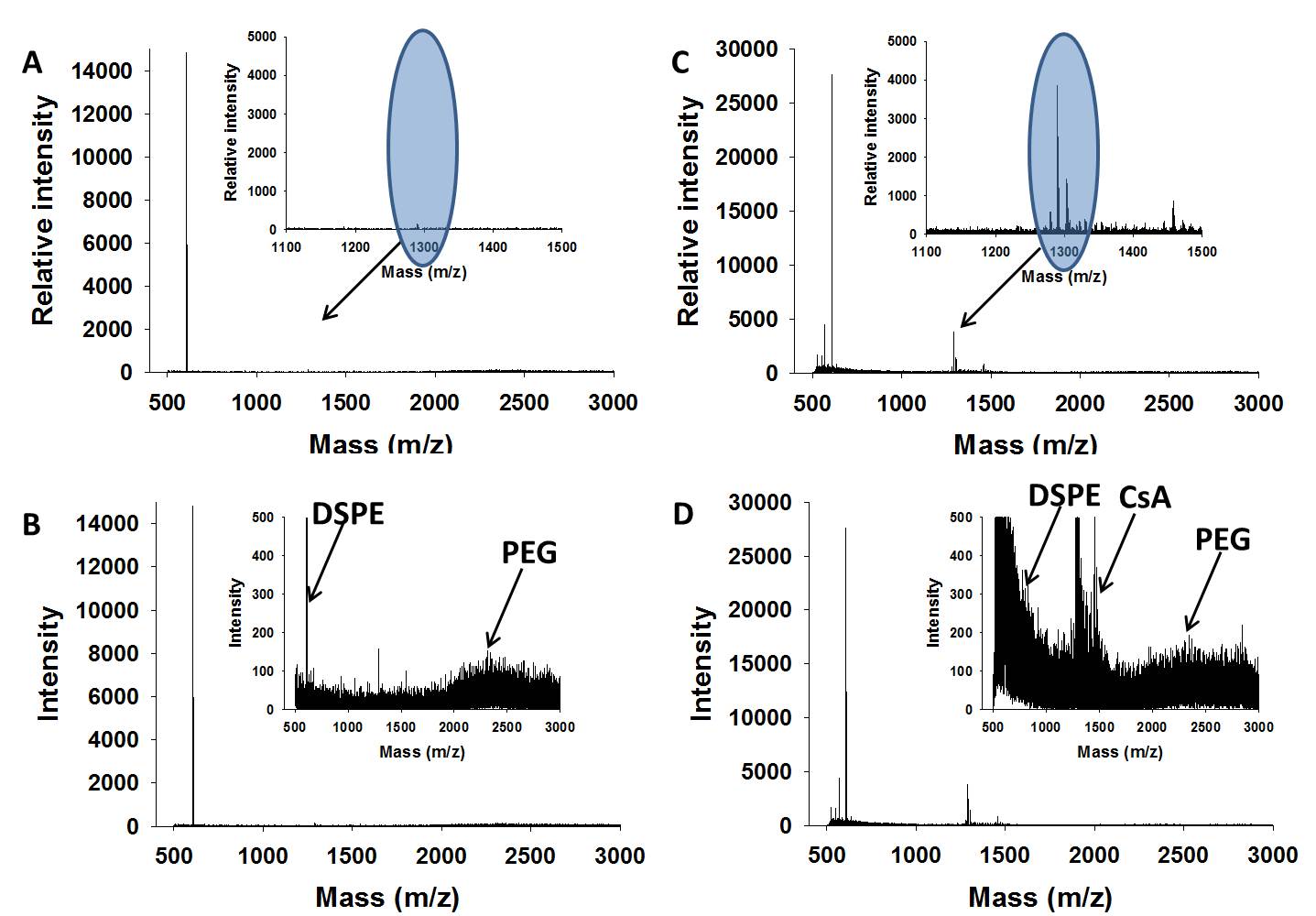


**Figure S3**. MALDI-TOF mass spectroscopy analyses of (A, B) DSPE-PEG-NHS and (C, D) DSPE-PEG-CsA. Appearance of the CsA peaks in DSPE-PEG-CsA suggest successful conjugation of the DSPE-PEG-NHS and CsA-NH_2_.


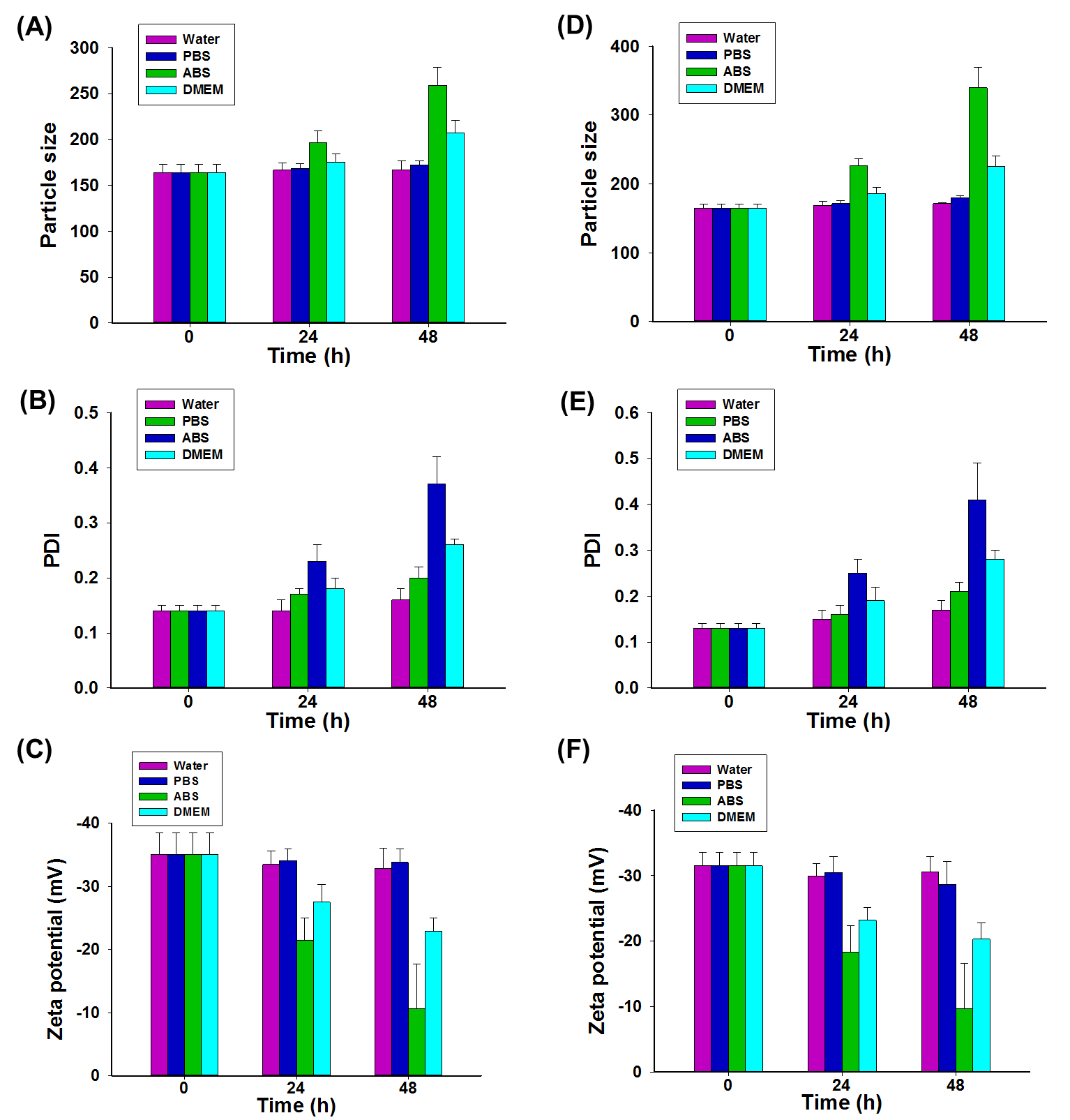


**Figure S4**. Stability evaluations of LMSN/BIR (A,B,C) and CLMSN/BIR (D,E,F) in water, PBS, ABS and DMEM upto 48 h.


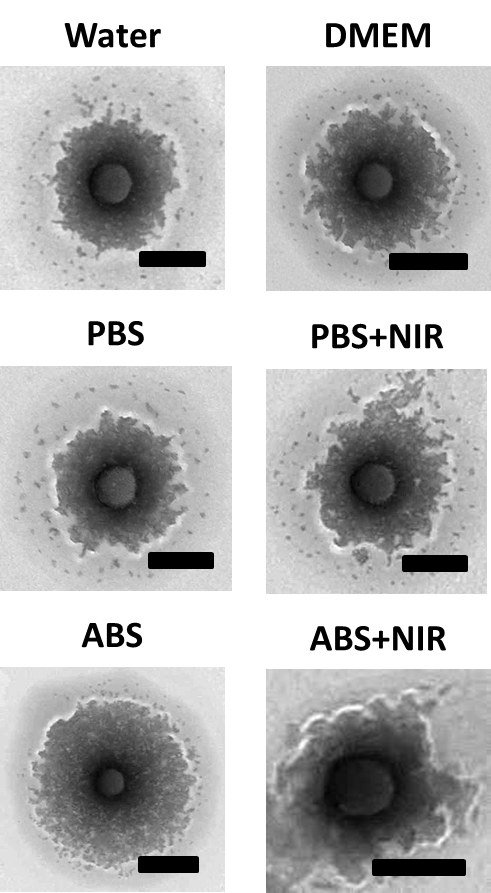


**Figure S5**. TEM images for the evaluation of morphological analysis of CLMSN/BIR in different media and under NIR laser irradiation (scale bar: 100 μm).


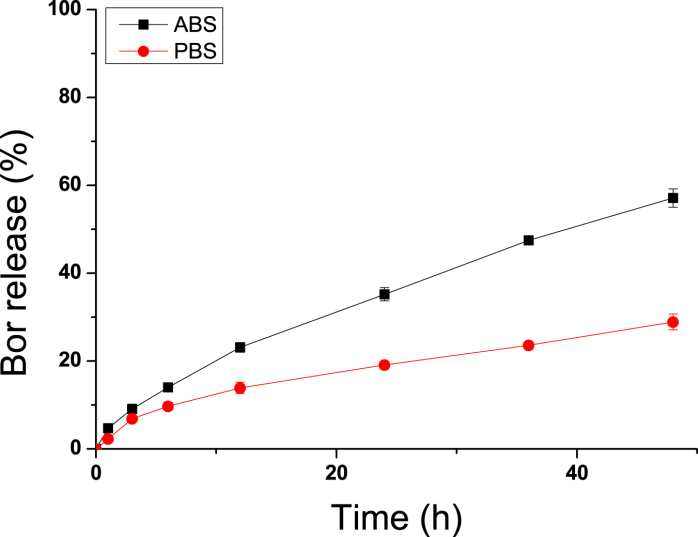


**Figure S6**. *In vitro* drug release profiles for Bor in ABS and PBS from LMSN/BIR.


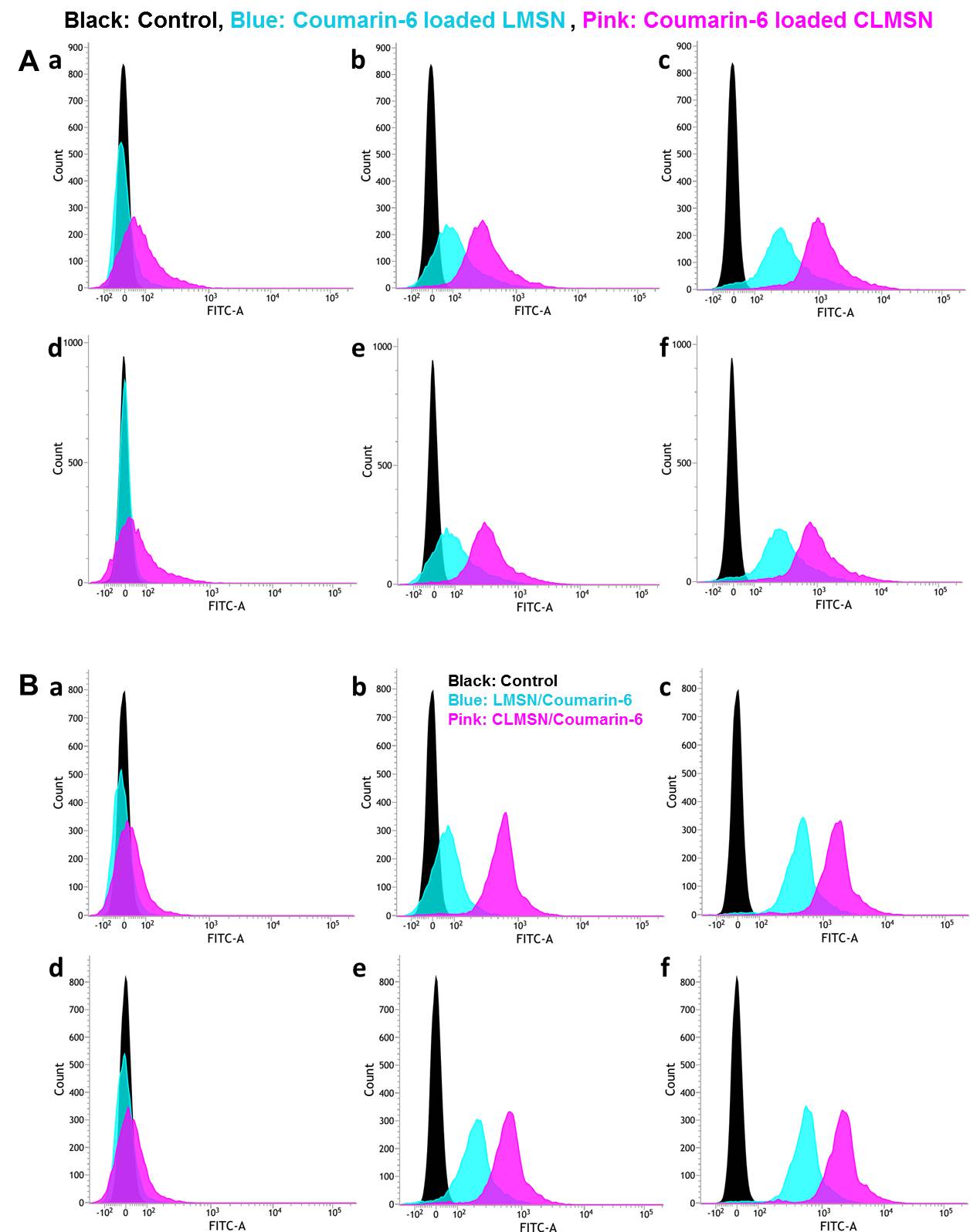


**Figure S7**. Comparative cellular uptake of coumarin-6 loaded LMSN and CLMSN in (A) PANC-1 and (B) MIA PaCa-2 cells in concentration- (a, b, c: 1, 3, 5 µg/mL) and time- (d, e, f: 30, 60, 90 min) dependent manners.


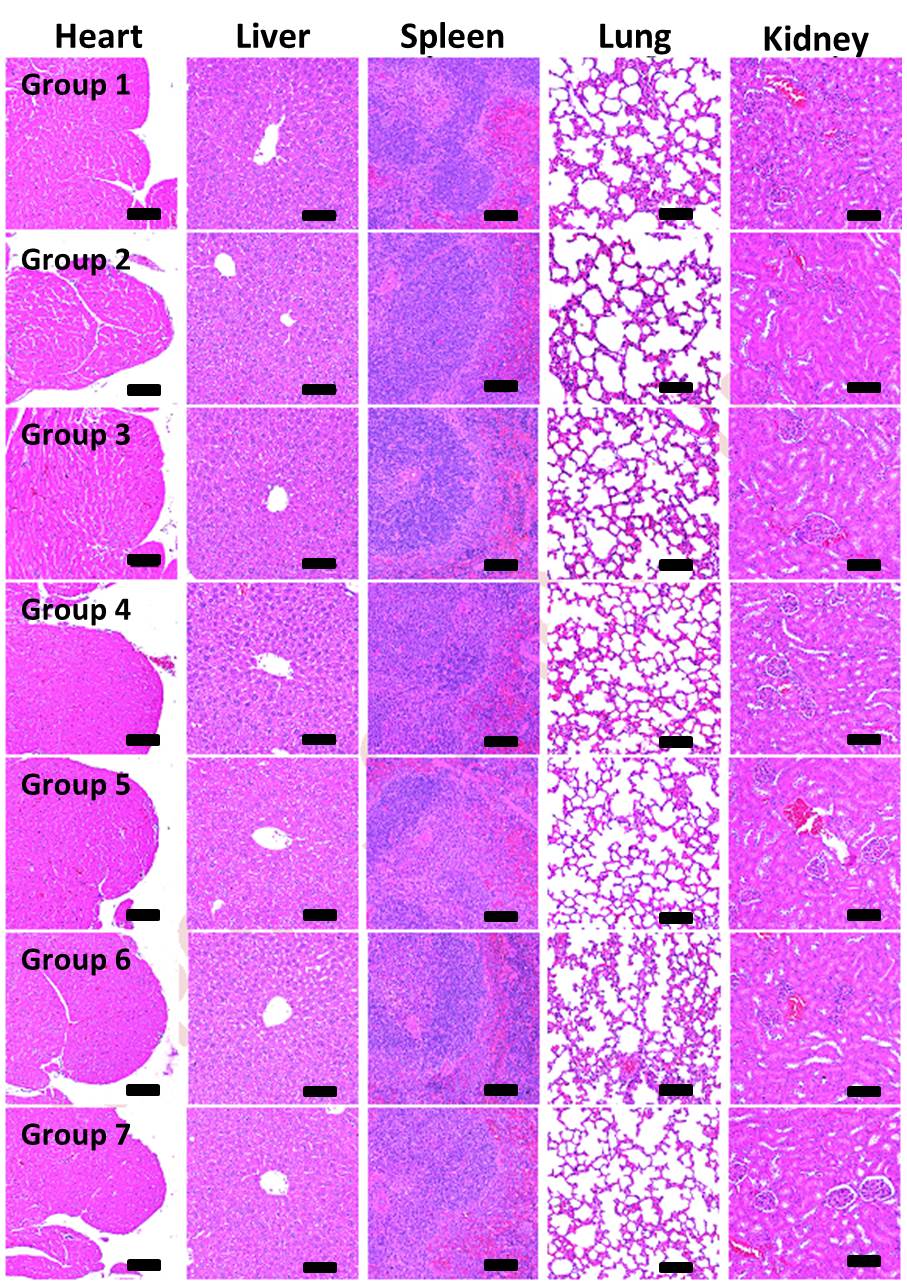


**Figure S8**. Representative organ histopathological images of mice from different groups: Group 1: Control, Group 2: BIR, Group 3: BIR+NIR, Group 4: LMSN/BIR, Group 5: LMSN/BIR+NIR, Group 6: CLMSN/BIR, Group 7: CLMSN/BIR+NIR (NIR exposure: 808 nm, 3.0 W/cm^2^, 5 min). Hematoxylin-eosin staining; scale bars: 120 μm.
